# Application Progress of the Single Domain Antibody in Medicine

**DOI:** 10.3390/ijms24044176

**Published:** 2023-02-20

**Authors:** Huaping Tang, Yuan Gao, Jiangyuan Han

**Affiliations:** 1 College of Life Science and Technology, Gansu Agricultural University, Lanzhou 730070, China; 2 Gansu Key Laboratory of Animal Generational Physiology and Reproductive Regulation, Lanzhou 730070, China

**Keywords:** sdAb, HCAb, nanobody, VHH

## Abstract

The camelid-derived single chain antibody (sdAb), also termed VHH or nanobody, is a unique, functional heavy (H)-chain antibody (HCAb). In contrast to conventional antibodies, sdAb is a unique antibody fragment consisting of a heavy-chain variable domain. It lacks light chains and a first constant domain (CH1). With a small molecular weight of only 12~15 kDa, sdAb has a similar antigen-binding affinity to conventional Abs but a higher solubility, which exerts unique advantages for the recognition and binding of functional, versatile, target-specific antigen fragments. In recent decades, with their unique structural and functional features, nanobodies have been considered promising agents and alternatives to traditional monoclonal antibodies. As a new generation of nano-biological tools, natural and synthetic nanobodies have been used in many fields of biomedicine, including biomolecular materials, biological research, medical diagnosis and immune therapies. This article briefly overviews the biomolecular structure, biochemical properties, immune acquisition and phage library construction of nanobodies and comprehensively reviews their applications in medical research. It is expected that this review will provide a reference for the further exploration and unveiling of nanobody properties and function, as well as a bright future for the development of drugs and therapeutic methods based on nanobodies.

## 1. Introduction

Antibodies, also called immunoglobulins (Ig), are at the core of an adaptive immune system. They have the ability to recognize and eliminate specific foreign substances from the body. Due to a natural ability to bind to target antigens, antibodies demonstrate great value in biomedicine and drug development [1,2]. To improve the clinical therapeutic efficacy, increase antigen recognition specificity, eliminate the immunogenicity and improve the affinity and stability of antibodies, monoclonal antibodies (mAb) were designed and produced. Since 1975, a significant amount of effort has been invested in monoclonal antibodies. Many monoclonal-antibody-targeted drugs were generated and used in biomedical research, disease treatment and clinical diagnosis [3]. As common biological macromolecules, monoclonal antibodies have had a particularly important pharmaceutical, commercial value over the past several decades [4], such as acting as an antibody–drug conjugate in the combination of CD147 monoclonal antibodies (CD147 mAb) and camptothecin polyphosphoester nanoparticles to effectively and precisely target hepatocellular carcinoma cells, improving the clinical, therapeutic effect on tumors [5]. However, at present, it is also very mature to obtain monoclonal antibodies for clinical disease treatment through phage library construction technology. Monoclonal antibodies are still macromolecules and have a complex internal structure. Multiple factors, such as a susceptibility to variable region folding, a low macromolecular permeability in tissues, and a high host immunogenicity, greatly hinder the development and application of antibody medicines [4].

A natural, small, functional antibody known as a heavy-chain antibody (HCAb) was first reported in camelid serum in 1993 [6]. Unlike conventional antibodies with a heterotetrametric structure, camelid-derived HCAb is devoid of light-chain polypeptides and lacks the first constant domain (CH1) in heavy-chains. Remarkably, the antigen-binding fragment in HCAbs contains only one single-variable domain. This domain is termed VHH and is also known as a single-domain antibody (sdAb) or nanobody (Nb) [7]. Uniquely, the strict, monomeric state of these nanobodies provides the ability to recognize and bind antigens independently after cloning and expression in vitro [8], such as the simplified, monomeric VHH-Fc antibodies [9,10] and small-scale, secretory VHH expression in Saccharomyces cerevisiae [11]. With a smaller biomolecule in the size range of 12~15 kDa and a higher affinity and stability, nanobodies, as promising agents in antibody engineering, have successfully attracted the attention of many biologists. For instance, humanized VHHs [12,13,14], the recombinant variable domains of heavy-chain-only antibodies that are made using a recombinant gene technique [15], have been used in cancer-targeting therapy. Obviously, the appearance of nano-antibody targeting drugs can greatly promote the development of antibody drugs in the field of tumor therapy when compared with traditional mAbs. CAR-T therapy brought a breakthrough in immuno-cancer treatment. Excitingly, research has demonstrated that VHH-based CAR-Ts disrupt the major limitations posed by the single-chain fragment variable (scFv) of an mAb; these limitations cover anti-idiotypic responses and pre-matured scFv aggregation, resulting in antigen-independent CAR-T exhaustion [16]. To summarize, nanobodies form the basis of a wide variety of applications, such as biological research, biomaterial development, medical diagnosis, immunotherapy and clinical trials. 

In this review, we comprehensively summarize the biomolecular structure of the HCAb and its VHH domain, mainly with respect to their biochemical properties, immune acquisition and phage library construction. We review some distinctive and interesting research and applications in the medical field, and expect that this review will provide a reference for the further exploration and unveiling of the properties and function of nanobodies, as well as providing a perspective for the development of drugs and therapeutic methods based on nanobodies.

## 2. Existing Forms of HCAb in Camelid Serum

**Figure 1:**
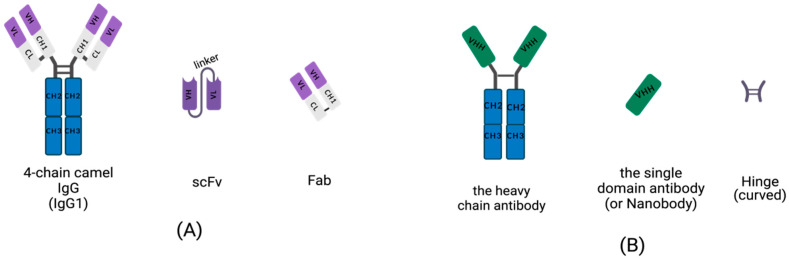
Schematic diagram for the primary structure of a conventional antibody, heavy-chain antibody (HCAb), nanobody, and small, recombinant antibody (This figure was created with BioRender.com, accessed on 27 March 2022). (**A**) A 4-chain camel IgG (IgG1), a class-conventional antibody in mammal serum, which consists of two light chains and two heavy-chains. The single light chain contains a variable region, VL, and a constant region, CL. The single heavy-chain contains a variable region, CL, and three constant regions, CH1, CH2 and CH3. The scFv is composed of a VH and VL pair-linked by an oligopeptide. A Fab fragment is formed by the first two domains of heavy-chain and light chain. (**B**) The single chain of HCAb contains two constant regions, CH2 and CH3 and a variable region, VHH, linked by curved hinge. VHH domain owns the smallest intact antigen-binding fragments, also known as single-domain antibodies (sdAbs) or nanobodies.

Conventional IgGs are tetramers consisting of two heavy-chains and two light chains (Figure 1A). The heavy-chain of an IgG includes three constant domains: CH1, CH2 and CH3, and a variable domain, the VH region. The light chain of an IgG contains a variable domain, VL, and a constant domain, CL (Figure 1A). Heavy-chains and light chains are linked by disulfide bonds between the VH and CH1. The variable regions, VH and VL, each contain three hypervariable, complementarity-determining regions (CDRs) that assemble to help form the correct antigen-binding site. Scientists are dedicated to manufacturing small, recombinant antibodies such as the single-chain fragment variable (scFv), which was generated by the concatenation of VH and VL via a linker oligopeptide using genetic engineering in vitro (Figure 1A) [17,18,19], and the fragment of antigen binding (Fab), which contains a light chain and the first two domains (VH and CH1 domain) of a heavy-chain, linked by hinges or disulfide bonds (Figure 1A) [20]. While the small, recombinant antibody has great potential for use in many fields, its development is limited by the insufficient affinity of VH and VL for antigens. 

Homodimeric antibodies are different from the conventional, heterotetrametric antibodies that naturally exist in the serum of camelids, including camels, alpacas and vicugnas. The homodimeric antibody has only two heavy-chains and is devoid of light chains; it is named HCAb (Figure 1B) [21]. Both conventional, 4-chain IgGs and HCAbs are present in camelid serum and possess the abilities of antigen-specific recognition and binding. Camelid HCAb is charactered by the absence of light chains and the lack of the CH1 in the heavy-chain. Therefore, the single-variable domains easily form a propensity for concave surfaces as active sites or receptor-binding pockets. HCAbs contain only two constant regions, CH2 and CH3, and a variable region, VHH, in each chain (Figure 1B). The terminal domain of a VHH is to engage with an antigen. This is the smallest functional, antigen-binding fragment in an HCAb, also known as an sdAb or a nanobody (Figure 1B). 

## 3. Molecular Structure of VHH

Muyldermans’s group constructed the VH/VHH dromedary germline database and compared the VH and VHH sequences of dromedary serum samples by PCR amplification. They found that the number of genes coding for VH and VHH of similar structures was almost the same in the camel genome, and that the VHHs evolved within a VH subgroup III [22]. Nevertheless, the variable domain sequences between the VH structure of the IgG and the VHH structure of the HCAb were an obvious distinction and mainly focused on the three high-variable regions, especially the amino acid sequence composition of the H3 loop with the largest change after rearrangement and the differences in the framework region, (FR) 2 [23].

The typical variable domain for the VHH structure of folded immunoglobulin is organized as nine β-strands, termed A-B-C-C’-C”-D-E-F-G and arranged in two sheets. The A, B, E, and D β-strands of one sheet lay one side and the C, C’, C”, G, and F strands lay on another. Three hypervariable (HV) regions cluster at one end of the domain: namely H1, H2, and H3. These regions are located in the loops that connect the B-C, C’-C”, and F-G strands, respectively. The three hypervariable regions are wrapped by the relatively conserved structure of the sheets, termed FR, and form a continuous surface with the capacity to bind to epitope, called the complementarity determining region (CDR) [24]. These three hypervariable regions are the most important parts for recognizing antigen-specific binding and have different functions. Compared with the HV loop of the conventional IgG1 VH domain, the sequence of the H1 loop is not classified, the H2 loop has a relatively canonical conformation and diverse structures, and the H3 loop has the largest difference in amino acid arrangement, sequence length, and structural change [25].

The longer H3 loop in the VHH domain of the HCAb may be due to the selection of multifunctional VHHs after the V-D-J rearrangement. With the expansion of H3, the entire HV loop also becomes longer, which can provide a sufficiently large antigen-interacting surface to better identify the epitope groove of the protein to combine with the antigen and antibody [26]. However, this specific antigen–antibody binding may also be due to the fact that the rearranged, autonomous VHH gene has a smaller molecule (independent of the appearance of VL domain, which can be seen in a conventional IgG1). This has not yet been proven by researchers with respect to molecular extended conformation kink folding and more complex molecular-recognition mechanisms [27]. Regardless, the expansion of the overall HV loop provides greater flexibility but also greatly reduces the stability of the internal structure of the VHH domain. Intriguingly, the extra interloop disulfide bond exists between the H1 and H3 loops. The interloop disulfide bond strengthens the extended HV loop structure and eliminates the flexibility brought by the longer H3 loop, which is beneficial for antigen binding [28]. 

Another distinction existing between the VHH domain of the sdAbs and the VH domain of the conventional antibody is the FR2 fragment. Researchers analyzed the VHH protein–antigen complex using X-ray crystallography and found that the hydrophobic amino acids in the VHH FR2 structure were replaced by the hydrophilic amino acids in the FR2 structure that cross-linked the VH-VL domain of conventional antibodies, which are Phe-42-Val, Glu-49-Gly, Arg-50-Leu, and Gly-52-Trp [29,30,31,32]. They explored the conformational space balance based on the hallmark amino acid residues of the sdAb VHH domain, thereby reconstructing the single-chain VHH domain. This can greatly improve the stability, solubility and biomolecule expression of nanobodies, which can be used as drugs and biosensors [33]. Reconstructing the nanobodies also demonstrated unique advantages such as the high affinity required to enable the specific targeting of antigen molecules, which is used to study and recognize target molecules in or on the surface of cells in cytochemistry [34].

## 4. Biochemical Features of the sdAb

### 4.1. Stability

Chemical inductions, such as a high concentration of guanidine hydrochloride (GuHcl) and urea, can lead to the reversible unfolding of antigens specifically bound to the CDR region and labile, refolded protein molecules. These can be achieved using far ultraviolet CD spectroscopy and surface plasmon resonance (SPR) for monitoring. In addition, the synergistic unfolding of the nanobody fragments was also observed by pressure induction. The above studies illustrate that the VHH fragment of HCAbs in camelid serum has a remarkably high conformational stability [35,36].

SdAbs can perform functions under high temperature exposure due to their ability to refold following heat denaturation. The VHH fragment of the HCAb in camel serum has a distinct thermo-reversible stability profile [37,38]. Most of the denatured VHH-R2 fragment population can be refolded at an extremely high temperature. These VHH-R2 molecules, induced by antigen–antibody production, are in an unfolded conformation before binding, indicating that the CDR region of the antigen recognition site in VHH has a non-canonical conformation, making the HCAb fragments fully thermally reversible [39,40]. The VHH fragments selected from the phage display library showed reversible thermal overlap with less aggregation via transient thermal denaturation [41]. Additionally, some efforts can further enhance the thermal stability of sdAbs by raising their melting temperature. Two common methods are: CDR grafting onto known, stable frameworks, and making point mutations to residues that have been identified to enhance stability from highly stable exemplars [42,43]. Notably, the insertion of a non-canonical disulfide bond between framework regions often yields an increase of 10–20 °C in melting temperature without considering the influence on affinity [41]. Evidence showed that the atypical Cys pairs generated in the VHH structure of the HCAbs via the replacement of Cys residues by Leu45 [41] in CDR-FRs produced a non-canonical disulfide linkage between CDR1-CDR3, endowing the VHH fragment with selective stabilization [44]. Additionally, increases in the net negative charge of proteins by chemical modification can increase the resistance of proteins to irreversible inactivation upon exposure to denaturing conditions and decrease the rate of protein aggregation, which is widely used in enzyme improvement [45]. It is also a potential method for improving the stability of a nanobody. 

Recently, molecular dynamics (MD) simulations were established to predict mutations affecting the thermal stabilization of sdAbs [46]. This approach estimated the relative stability for a set of sdAbs for which both crystal structures and the melting transition temperature (Tm) were available. Correlations between the fraction of native atomic contacts (denoted by Q) and the experimental Tm values were generated based on computing the Q for the sdAbs at three different temperatures [46]. This approach provided valuable guidance for identifying potential mutation sites that are unlikely to be easily ascertained by any other means, which is useful for modeling to direct t experiments [41]. 

### 4.2. Affinity (Solubility)

The hydrophilicity of an antibody’s light-chain surface [47], which are adept at absorbing the hydrophobic surfaces of nonionic surfactants [48], is very important for the development of antibody drugs. The variable heavy-chain domain of human antibodies arises from recombination, and the dispersed proteins re-aggregate under physical conditions such as heat and acid. The characteristics of protein aggregation were studied by analyzing the CDR sequence of the phage display, which is convenient for the research, development, and application of VHs libraries [49]. Furthermore, researchers analyzed the CDR-region amino acid residues through the phage display of a human VH library with a constrained CDR3 loop. This yielded non-aggregating VHs, and the non-aggregative VHs almost existed in the non-canonical CDR1-CDR3 loop at the acid isoelectric point (PI). The PI distribution analysis of the human VH sequence and the Camelidae rearranged VHH sequence revealed that the VHH–acid ratio was higher. Therefore, the design of VHs libraries with non-canonical and non-aggregation intra-CDR disulfide bonds can greatly improve the solubility of the VH domain [50]. In addition, as it is known that proteins behave with minimal solubility at the isoelectric point, increasing the negative charge of the nanobodies in CDR sequences via mutation or modification can also prevent protein aggregation upon denaturation by, for instance, heat or acid [51].

In the VHH of an HCAb, the CDR-H3 region has a larger frequency variation, ranging from 10 to 24 amino acids [52], while the CDR3 fragment of the human VH domain has only 11.6 amino acids [53]. Therefore, compared to conventional Ig antibodies, the variable domain structure of the camelid sdAb has relatively longer CDR3 fragments. As can be seen in the 2.5A resolution representation of the complex crystal structure of the CDR3 region, CDR3s more readily provide antigen-binding sites on their surface grooves, and other parts of CDR3s can also penetrate deeply into the active site of a lysozyme complex. These unique characteristics of the camel VHH fragment determine its higher affinity, or solubility, and have certain anti-aggregation properties [54,55,56].

### 4.3. Epitope Recognition of Multivalent Bispecific Antibody of Nbs-Based Minimal Size

In recent decades, bispecific antibodies (BsAbs), as intermediate-sized biomolecules, have attracted extensive attention as therapeutic tools in the field of medicine [57,58]. Although BsAbs have excellent pharmacokinetics, they demonstrate extremely low penetration in renal tissue [59]. It is necessary to develop molecular binding mechanisms for soluble, double and trivalent conjugates to improve the retargeting function of effector T cells to tumors [60,61].

The VH and VL domains of the conventional antibody IgG were covered via two independently functioning VHH domains of the HCAb, assembled into bispecific VHH-IgG and combined with a heavy-chain heterodimerization technology to produce monovalent, bispecific, IgG-like antibodies [62]. Pekar’s team used the strand exchange engineering domain (SEED) technology to optimize the heterodimer of the VHH, which was inserted into the Fc backbone to generate trivalent, specific, IgG-like compounds. The generation of multivalent compounds can use ten amino acid linkers to connect the binding sites so that a VHH is engrafted onto a constant region, CH1, while the other VHH-based paratope is replaced on the CH1 region of the light chain, making full use of the biochemical properties of nanobodies without reducing the nanobodies’ affinity to construct a tetravalent, bispecific, IgG-like molecule [63].

Antigen-specific nanobodies are obtained by animal immunization and yeast antibody coupling agent display technology. The exogenous VHH is non-immunogenic [64], and humanized nanobodies are developed by altering the differentially marked amino acids of the VHH fragment [65]. 

## 5. Generation of VHH Libraries

The VHH repertoire of the dromedary HCAb is a biological protein molecule, produced through the stimulation of the B cells of immune animals with an antigen. The high-affinity VHH library generated via specifically combining two cognate antigens is also called the immune library. A unique advantage of maturated, in vivo immune libraries is the relatively high quality of VHH sequences to avoid the immunogenicity of antibodies [66,67]. In addition, there are two other types of VHH libraries (or nanobody libraries), i.e., naïve libraries [68] and synthetic libraries [69]. 

Some researchers immunized camelids with an anti-DNA mouse mAb, which can cause an anti-idiotypic response and activate the humoral immune system, generating VHHs by constructing phage libraries [70]. However, the molecular mimicry approach makes it difficult to obtain syngeneic anti-Ids using anti-idiotypics. Some other studies prepared variable domain fragments of useful, single-domain recombinant antibodies by immunizing llama libraries as an alternative to conventional, monoclonal antibody libraries [71]. Using the immunized alpaca B lymphocytes for VHH libraries, the construction has the unique properties of high titers of intact, single-domain antigen-cognate binders and an ease of affinity. These immunoselected, recombinant nanobody libraries are well expressed. Antigen-binding nanobody (chromobody) technology for the identification and tracking of antigens in living cells is a new and mature tool for the selection and identification of VHH libraries [72,73].

**Figure 2:**
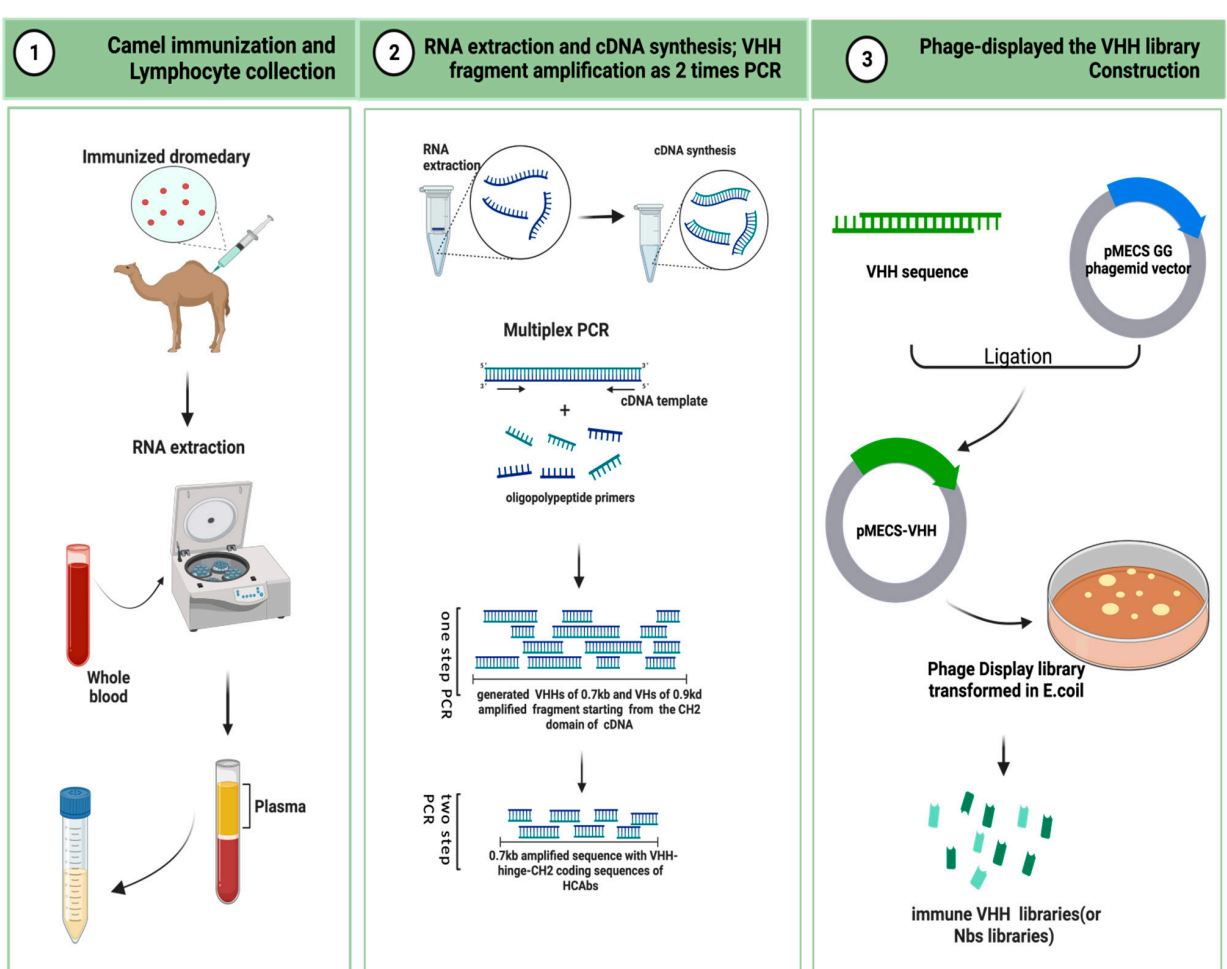
Schematic representation to generate immune VHH libraries (This figure was created with BioRender.com, accessed on 8 August 2022).

Here, we describe the critical steps in the preparation of an immunized VHH library. As is shown in Figure 2, camels are immunized to obtain a B lymphocyte blood sample in vivo, and the library is then cloned for display on bacteriophage particles in *E. coli* [74]. A single-domain VHH library with a minimal, fully engineered, antigen-binding fragment from a camelid and strict-monomer state, heavy-chain-only immunoglobulins with high-quality VHH sequences are responsive to specific antigen binding agents and small library sizes (10^6^–10^8^). Furthermore, affinity-matured, immune nanobody repertoires from these high-standard, immunized, in vivo procedures can better reach pre-specified epitopes and adapt to the immunogenicity of humanized antibodies for the preparation of bioengineered sdAbs [66,75]. 

### 5.1. Camel Immunization and Lymphocyte Collection

A healthy adult camel was injected with the antigenic protein of a mixed-compound adjuvant once every two weeks, four to eight times in two months. Camels can use less than approximately 200 mg of mixed adjuvant per injection. The anticoagulated blood of 50 mL from an immunized animal were collected after eight injections. B-lymphocytes were isolated from the immunized camel serum for total RNA extraction, and stored at −80 °C for later use. For long-term storage, the samples were placed in liquid nitrogen [76,77]. 

### 5.2. RNA Extraction and cDNA Synthesis: VHH Fragment Amplification from Two-Step PCR [78] 

The total RNA was extracted from approximately 10^7^ B-lymphocyte apheresis samples for cDNA synthesis. Oligomeric polypeptide primers were designed based on the single-chain variable domain sequences of the Ig antibodies, and PCR amplification was performed to obtain the VHH gene fragments.

The two-step PCR amplification began from the CH2 domain of an IgG cDNA template. It yielded two distinct PCR states of approximately 0.7 kb and 0.9 kd. The 0.7 kb amplification product was derived from the mRNA coding sequence of the variable domain of the homodimer antibody single chain, which required further agarose gel purification. The purified product was used as a template for PCR amplification again, and only the VHH sequence of the HCAb was obtained. 

### 5.3. Phage Display of the VHH Library Construction [76]

The pMECS GG (a pUC-derived bacteriophage with an F1 responsive source and an IPTG-inducible Plac promoter expression) or another suitable phage display vector were used to ligate the gene fragments of the VHHs to the two-step, amplified PCR products to obtain affinity-matured, robust, recombinant VHH repertoires. The VHH gene sequence ligated to the recombinant vector was again purified and then transformed in *E. coli* TG1 to generate a small library (10^7^ to 10^8^) of high titer of target-specific binders:s the VHH library. 

## 6. Nanobody Applications in Medical Research

### 6.1. Molecular Diagnostic Potential of Nanobodies

As an important part of modern medicine, molecular imaging can provide precise tracing and reliable information at the cellular or molecular level through radio-labeled antibodies [79]. Tumor molecular imaging promotes the early diagnosis and treatment of tumors, and can effectively detect biomarkers in the tumor microenvironment [80]. However, the molecular lesions of advanced tumors are complex, and conventional molecular imaging cannot detect scattered lesions. As smaller, intact, functional antigen binding agents with a small size (12–15 kDA), nanobodies demonstrate strong tissue penetration and high affinity and stability, revealing treatment pharmacokinetics and pharmacodynamics compatible with short-lived radioisotopes. They are more suitable for tracking advanced tumors [81,82,83].

Nanobody-based, non-invasive molecular imaging techniques have been widely used as cancer cell markers. One such example is ^99m^Tc, a radio-labeled, anti-epidermal growth factor receptor (EGFR) nanobody that can detect EGFR-expressing cancer cells with higher accuracy and visualization [84]. As imaging immune checkpoint tracers for infiltrating macrophage immune diseases, nanobody-based imaging monitoring can identify immune response patterns, predict responses during therapy, and trace the biological processes of immune system activation, thereby improving immunotherapy [85]. More molecular imaging techniques based on nanobody development are shown in Table 1. 

### 6.2. Clinical Trials of Humanized Nanobodies

In the FR characterization of camelid and human family counterparts, the major camelid immunoglobulin, light-chain, lambda variable region (IGLV) families 1 and 3 are 81 to 91% identical to their human counterparts, respectively. Human immunoglobulin heavy-chain variable region (IGHV) families 1 to 3 have FR sequence identities of 92 to 95% compared to camelids, with an overall identity ranging between 61 and 91%. By analyzing the FR framework and canonical CDR structures of the camelid, homodimeric antibody and its human counterparts, the V family gene sequences were matched and a high degree of homology was revealed. Based on highly homologous and remarkable physicochemical properties, camelid nanobodies can effectively deal with the immunogenicity brought by humanization and can provide a variety of clinical, therapeutic antibody transformations [99]. Identifying amino-acid-residue binding sites on the surface of the VHH structure would be help to humanize the nanobodies and further improve their stability, immunity, solubility, and affinity for antigen binding and develop clinical nanobody therapeutic platforms [100].

According to the unique properties of nanobodies, some reports synthesized an anti- human EGFR2 (HER2) nanobody and established animal models. The study testified that the anti-HER2 nanobody showed high, specific tumor uptake but low kidney uptake, demonstrating that anti-HER2 nanobodies have good sensitivity for tumor detection with non-toxicity in a preclinical, validated therapy [101]. Some nanobody-based antibody drugs have been used in preclinical and clinical applications, such as the ^68^Ga-HER2-nanobody used in Phase 1 of HER2-positive breast carcinoma, which demonstrated obvious efficacy after PET/CT assessment [102]. More clinical trials are available in Table 2.

### 6.3. Nanobodies as a Tumor Microenvironment 

#### 6.3.1. Nanobodies and Cellular Transmembrane Proteins’ EGFR Binding 

In cancer therapy, links between nanobodies and cytokines form transmembrane, protein-bound nanobodies or soluble adhesives. Nanobodies are driven from the variable domain of the IgG1 single-heavy-chain-only antibody, and the binding sites on the surface of the variable domain can specifically recognize cognate–antigen [22]. Due to the small size and ease of affinity of the natural, in vivo, multifunctional nanobodies, nanobody-based, conjugated protein molecules are also relatively small and can penetrate the vasculature to reach the target tumor cells, interfering with cell signal transduction and inhibiting tumor cell proliferation [103].

**Figure 3:**
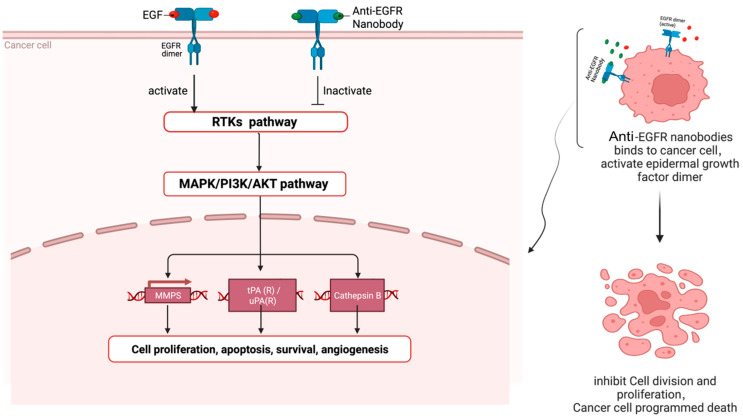
When anti-EGFR nanobodies reach the surface of cancer cells, anti-EGFR nanobodies can compete with EGF to bind EGFR dimers, target cancer cells and inactivate intracellular receptor tyrosine kinase (RTK) signaling pathway, causing cascade reactions of downstream pathways, thereby inhibiting the proliferation of cancer cells (This figure was created with BioRender.com, accessed on 8 August 2022).

For example, the anti-EGFR nanobody can inhibit tumor cell proliferation by inhibiting the intracellular tyrosine kinase signaling pathway network (Figure 3) [104]. When human epidermal growth factor (EGF) binds to EGFR, EGFR and its relatives, the ERBB family members, have mutations or increased expression, which will trigger a cascade reaction of upstream and downstream signaling pathways in the ERBB signaling pathway, especially the intracellular tyrosine kinase signaling pathway, eventually becoming various malignant tumors [105].

Laeremans’s group produced an anti-EGFR nanobody via the immunization of camels with EGFR-containing cell preparations, the isolation of mRNA from the camel serum and the transcription into cDNA, and separating the variable domain fragments of the camel HCAb by gel electrophoresis, and displaying it in a bacteriophage system [106]. Additionally, bivalent and trivalent specific nanobodies were also synthesized to improve the affinity of the anti-EGFR nanobody. The inhibition of EGF-induced cell proliferation was then conducted in a cell model [107]. The study found that due to their small size (12~15 kDa), the natural “immune” nanobody could easily be formatted after binding to EGF, quickly passed through the blood, and only had a short residence time at the target, which is not suitable for cancer treatment. However, the improved, bivalent, anti-EGFR nanobody used albumin fusion binding to the nanobody unit, resulting in a bifunctional nanobody, exhibited enhanced penetration, stimulated the receptor tyrosine kinase activity, and prohibited the transduction of the EGFR signaling network [108]. Bispecific antibodies can enhance the binding of EGFR and nanobodies, guide the recruitment of T cells to the antibody, and have a stronger therapeutic effect to inhibit tumor cell proliferation. In addition, the renal permeability and in vivo pharmacokinetics of the “immune” nanobodies were also improved [109].

#### 6.3.2. Nanobodies and Extracellular Target CXCR7 Binding 

CXC chemokine receptors (CXCRs) 4 and 7 are both members of the seven-transmembrane domain G protein-coupled receptor (GPCR) subfamily that can bind to the extracellular chemokine ligand, CXCL12, and elicit complex immune responses in vitro or in vivo [110]. There is a balance between G-protein- and β-arrestin-mediated signaling pathways; as an agonist, CXCR7 is not coupled to the G protein, and can transmit signals through β-arrestin [111]. The β-arrestin of cytoplasmic adaptor proteins can be driven by the binding of chemokine ligands to the protein molecules’ CXCR7, which promotes the uptake of CXCL12 by the chemokine receptor CXCR7, thereby causing the high expression of CXCR7 receptors and causing the formation and metastasis of tumors. Actually, the overexpression of CXCR7 is related to many tumors from various origins, including the lung, breast, brain, prostate and kidney [112,113,114].

**Figure 4:**
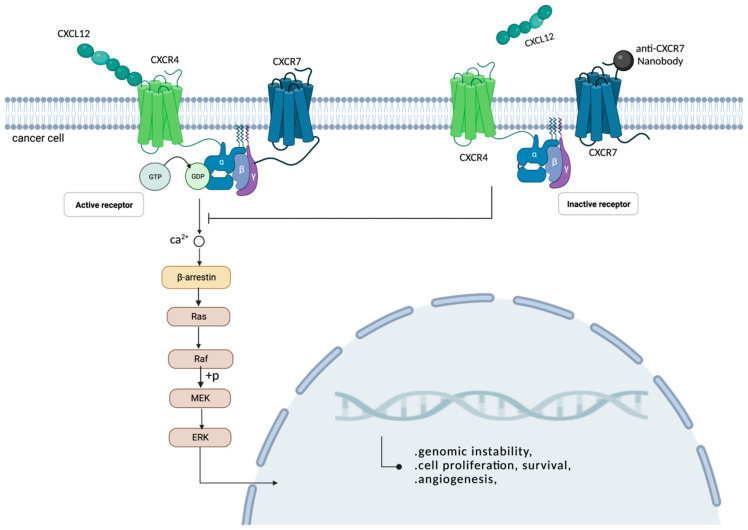
As an extracellular homeostasis chemokine, CXCL12 can bind to receptors CXCR4, forming CXCR4/CXCR7 homodimers, and activate intracellular signaling responses. When anti-CXCR7 nanobodies interact with receptors CXCR7, the binding of CXCL12 to the receptor CXCR4 is blocked, and the G-protein-coupled receptor cannot be activated, thereby inhibiting the intracellular calmodulin response, stopping the recruitment of β-arrestin in the cytoplasm, preventing downstream signaling pathways and inhibiting tumor cell proliferation (This figure was created with BioRender.com, accessed on 8 August 2022).

The CXCR7 nanobody, also called the VHH-derived llama immunoglobulin single variable domain, is a small-molecule ligand with a selective microaffinity characteristic that has a significant action in immune diseases and tumor-intervention therapeutics. This VHH-derived nanobody can compete with the ligand CXCL12 to bind the chemokine receptor CXCR7, antagonize the CXCL12 chemoattractant effect and inhibit the CXCR4/7-mediated signaling, hereby inhibiting the proliferation of tumors (Figure 4) [115,116].

Maussang’s team adopted two strategies to immunize llamas to ensure that highly selective nanobody-biologicals were produced that would compete for extracellular-membrane-binding receptors and could correctly target chemokine receptor CXCR7: namely, the CXCR7-targeting nanobody [117]. CXCR7 was expressed in human kidney cells (HEK293), which were then used to immunize llamas, and 78 unique, CXCR7-specific nanobodies were identified from 45 different lineages of B lymphocytes. These B lymphocytes are capable of producing the single-variable region of the naturally occurring, heavy-chain-only camelid immunoglobulin antibody. One or two N-terminal special CXCR7s and one C-terminal special, human-serum-albumin building block were used to construct bivalent and trivalent nanobodies. Finally, two rounds of PCR amplification were used for screening, targeting the CXCR7 nanobodies. Based on this, the research team developed and characterized a highly selective and functional CXCR7-targeting nanobody [117]. 

Many GPCRs have extremely complex and conserved conformational changes across species [118]. This is not conducive to the production of medical, therapeutic antibodies from the immune library. The nanobody platform, a novel biological tool for effectively handing difficult drug targets, is a class of valuable platform developing antibodies as functional antagonists of GPCR-mediated signaling in tumor therapy and other research applications [119]. A synthetic yeast display library can effectively screen antibody fragments that regulate receptors which are active in vitro and in vivo, paving the way for the drug therapy of GPCRs [120]. 

## 7. Conclusions and Prospective Research

The nanobody is the variable domain of heavy-chain-only antibodies, also called the sdAb. Since the discovery of the nanobody, due to its natural advantages, it has attracted the extensive attention of researchers and is considered to have a bright future in many fields, such as biomedicine and bioengineering. There have been many breakthroughs in nanobody research [121,122,123,124]. Compared with traditional antibodies, the nanobody has not only better physiochemical properties, such as a strict monomer state and high stability and solubility, but also has mature, multiplex PCR screening and a phage display technique. Therefore, for small molecules, nanobodies share new possibilities in many biomedical applications, including molecular imaging, clinical antibody drug development, and the use of humanized, tumor-targeting drugs in vitro. We comprehensively summarize the biomolecular structure of the nanobodies and their VHH domain, their major physiochemical properties, immune acquisition and phage library construction, and reviewed some distinctive and interesting research and applications in the medical field. We expect that this review will provide a reference for the further exploration and unveiling of the properties and function of nanobodies, as well as provide a perspective for the development of drugs and therapeutic methods based on nanobodies.

The nanobody present in camel B lymphocytes was obtained after antigen immunization. In addition to the homologous dimer antibody containing the nanobody, there are also a large number of homologous tetramer antibodies in camel serum after immunization. Although phages are a mature, recombinant antibody fragment technology, after two PCR amplifications, VHH libraries with a high stability and high affinity were obtained. However, due to their small size, which resulted in a short blood retention time and low immunogenicity [125], the VHH fragments obtained from the immunized camel serum made it difficult to achieve a subsequent clinical, therapeutic effect of humanized nanobodies. Although numerous studies have broken through this problem and achieved results [126,127], further in-depth research is required on how to make the efficient conversion of a small amount of VHH fragments for the subsequent clinical application of nanobodies.

Nanobodies are the smallest antigen-binding functional fragments, based on a small molecular weight, ease of identification, and the database knowing the VHH gene fragments. At the CDR region site that binds to the ligands of a specific structure, a binding-mutated site was built with loops that is easily varied and flexible. Based on the nanobodies’ physical properties of affinity and stability, the recombinant nanobodies are able to better pass through the blood–brain barrier, meaning that the nanobodies’ biologics can be targeted to the target lesions and achieve clinical, therapeutic effects. Compared with traditional antibodies, it is longer that the CDR3 region of the variable domain of the single, heavy-chain antibody present in camel serum, and substituted amino acid residues have been reported [128]. However, the folded conformation of the rearranged V-D-J fragment is not clear, and the function of the disappearing light-chain in the B-cell stage is also worth investigating.

## Figures and Tables

**Table 1:** Nanobody development for preclinical molecular diagnostics (Data sources for clinical trials: https://clinicaltrials.gov/, accessed on 9 August 2022).

Target	Agent	Title	Clinical Trials: Stage and Status/References
HER2	^99m^Tc/^188^Re	HER2 Expression Detection and Radionuclide Therapy in Breast Cancer Using ^99m^Tc/^188^Re Labeled Single Domain Antibody	Clinical, NCT04674722 (Early Phase 1)
	^99m^Tc-NM-02	HER2 Expression Detection in Breast Cancer Using ^99m^Tc-NM-02	Clinical, NCT04040686 (Early Phase 1)
	^99m^Tc-7C12	Correlation between EGFR-Nbs uptake and tumor burden	[86]
HER3	^89^Zr-MSB0010853	^89^Zr-MSB0010853 monitor Human EGFR3-Specific Tumor Uptake and Biodistribution	[87]
PD-L1	[^99m^Tc]-NM-01	PD-L1 Expression in Lung Cancer	Clinical, NCT04992715 (Phase 2)
AlbudAb	^68^Ga-THP-APN09	PD-L1 Targeting Nanobody Probe for PET Imaging of Solid Tumor	Clinical, NCT05156515 (Not Applicable)
	^18^F-B3, ^18^F-A12, ^64^Cu-B3	PD-L1 for an activated-independent adipocytes biomarker	[88]
	^68^Ga-NOTA-Nb109	^68^Ga-NOTA-Nb109 monitor cisplatin (CDDP) induced NCLH1299 cells PD-L1 expression	[89]
	^89^Zr-Df-KN035	^89^Zr-Df-KN035 monitor PD-L1 expression in nude mice bearing LN229 xenografts, and used to evaluate the whole-body biodistribution in non-human primates (NHPs)	[90]
	^99m^Tc-C3, ^99m^Tc-C7, ^99m^Tc-E2, ^99m^Tc-E4	The nanobody imaging is used assess murine PD-L1 expression in syngeneic tumor models	[91]
	^89^Zr-GSK3128349	A Positron Emission Tomography (PET) Imaging Study to Investigate the Biodistribution and Clearance of an Albumin Binding Domain Antibody (AlbudAb) GSK3128349 in Healthy Male Subjects	Clinical, NCT02829307 (Phase 1)
CD20	^68^Ga-9079	the Anti-CD20 Nb for targeted radionuclide therapy of human Non-Hodgkin Lymphoma	[92]
CD38	^68^Ga-NOTA-Nb1053	ImmunoPET imaging of multiple myeloma with ^68^Ga-NOTA-Nb1053	[93]
CD8	^89^Zr-VHH-X118	Predicting the Response to CTLA-4 Blockade by Longitudinal Noninvasive Monitoring of CD8 T Cells.	[94]
	^68^Ga-NOTA-SNA006	Human CD8^+^ T Cells with ^68^Ga-Labeled Nanobody Diagnostic	[95]
CEA	^99m^Tc-NbCEA5	Tumor Targeting of a Humanized, Grafted Nanobody in Mice Using Pinhole SPECT/Micro-CT	[96]
PSMA	^111^In-JVZ007	A ^111^In-Labeled Anti-Nanobody for Prostate Cancer	[97]
HGF	^89^Zr-1E2, ^89^Zr-6E10	Nanobodies Targeting the Hepatocyte Growth Factor	[98]

**Table 2:** Study progress for nanobodies or single-domain antibodies in preclinical and clinical trials (data sources for clinical trials: https://clinicaltrials.gov/, accessed on 9 August 2022).

Rank	NCT Number	Title	Phases
1	NCT03224702	First-in-Human Trial of Anti-ADAMTS-5 Nanobody in Healthy Volunteers	Phase 1
2	NCT03583346	Multiple Ascending Doses (MAD) of Anti-A Disintegrin and Metalloproteinase With Thrombospondin Motifs-5 (Anti-ADAMTS-5) Nanobody in Participants With Knee Osteoarthritis (OA)	Phase 1
3	NCT02156466	Multiple Ascending Dose Trial of MSB0010841 (Anti-IL17A/F Nanobody) in Psoriasis Subjects	Phase 1
4	NCT03881761	CD19/20 Bispecific Nanobody-derived CAR-T Cells in B Cell Lymphoma	Phase 1
5	NCT01151423	Study to Assess Efficacy and Safety of Anti-von Willebrand Factor (vWF) Nanobody in Patients With Acquired Thrombotic Thrombocytopenic Purpura (aTTP)	Phase 2
6	NCT04489862	αPD1-MSLN-CAR T Cells for the Treatment of MSLN-positive Advanced Solid Tumors	Early Phase 1
7	NCT01374503	First in Man Study of ALX-0651, a Nanobody Inhibiting CXCR4	Phase 1
8	NCT01284569	Study to Assess Safety and Efficacy of Anti-Interleukin 6-receptor (IL6R) Nanobody in Rheumatoid Arthritis (RA) Patients	Phase 1|Phase 2
9	NCT03664661	BCMA-CAR-T in Relapsed/Refractory Multiple Myeloma	Phase 1
10	NCT01259765	Llama Antibody, Rotavirus Diarrhoea, Children	Phase 2
11	NCT04469725	KN046 (a Humanized PD-L1/CTLA4 Bispecific Single Domain Fc Fusion Protein Antibody) in Subjects With Thymic Carcinoma	Phase 2
12	NCT04126590	Phase I Study of KN044 in Locally Advanced/Metastatic Solid Tumors	Phase 1

## Data Availability

Not applicable.

## References

[1] Alfaleh M.A., Alsaab H.O., Mahmoud A.B., Alkayyal A.A., Jones M.L., Mahler S.M., Hashem A.M. (2020). Phage Display Derived Monoclonal Antibodies: From Bench to Bedside. Front. Immunol..

[2] Fernandes J.C. (2018). Therapeutic application of antibody fragments in autoimmune diseases: Current state and prospects. Drug Discov. Today.

[3] Shepard H.M., Phillips G.L., Thanos C.D., Feldmann M. (2017). Developments in therapy with monoclonal antibodies and related proteins. Clin. Med..

[4] Nelson P.N., Reynolds G.M., Waldron E.E., Ward E., Giannopoulos K., Murray P.G. (2000). Monoclonal antibodies. Mol. Pathol..

[5] Tian H., Huang Y., He J., Zhang M., Ni P. (2021). CD147 Monoclonal Antibody Targeted Reduction-Responsive Camptothecin Polyphosphoester Nanomedicine for Drug Delivery in Hepatocellular Carcinoma Cells. ACS Appl. Bio Mater..

[6] Hamers-Casterman C., Atarhouch T., Muyldermans S., Robinson G., Hamers C., Songa E.B., Bendahman N., Hamers R. (1993). Naturally occurring antibodies devoid of light chains. Nature.

[7] Yu X., Xu Q., Wu Y., Jiang H., Wei W., Zulipikaer A., Guo Y., Jirimutu, Chen J. (2020). Nanobodies derived from Camelids represent versatile biomolecules for biomedical applications. Biomater. Sci..

[8] Muyldermans S. (2021). Applications of Nanobodies. Annu. Rev. Anim. Biosci..

[9] De Greve H., Virdi V., Bakshi S., Depicker A. (2020). Simplified monomeric VHH-Fc antibodies provide new opportunities for passive immunization. Curr. Opin. Biotechnol..

[10] De Greve H. (2022). Production of Designer VHH-Based Antibodies in Plants. Methods Mol. Biol..

[11] Harmsen M.M., van Hagen-van Setten M., Willemsen P.T.J. (2022). Small-Scale Secretory VHH Expression in Saccharomyces cerevisiae. Methods Mol. Biol..

[12] Zhong W., Lu Y., Ma Z., He Y., Ding Y., Yao G., Zhou Z., Dong J., Fang Y., Jiang W. (2022). Development of a Humanized VHH Based Recombinant Antibody Targeting Claudin 18.2 Positive Cancers. Front. Immunol..

[13] Chi H., Wang L., Liu C., Cheng X., Zheng H., Lv L., Tan Y., Zhang N., Zhao S., Wu M. (2022). An Engineered IgG-VHH Bispecific Antibody against SARS-CoV-2 and Its Variants. Small Methods.

[14] Li J., Deng Y., Zhang W., Zhou A.P., Guo W., Yang J., Yuan Y., Zhu L., Qin S., Xiang S. (2021). Subcutaneous envafolimab monotherapy in patients with advanced defective mismatch repair/microsatellite instability high solid tumors. J. Hematol. Oncol..

[15] Sun S., Ding Z., Yang X., Zhao X., Zhao M., Gao L., Chen Q., Xie S., Liu A., Yin S. (2021). Nanobody: A Small Antibody with Big Implications for Tumor Therapeutic Strategy. Int. J. Nanomed..

[16] Safarzadeh Kozani P., Naseri A., Mirarefin S.M.J., Salem F., Nikbakht M., Evazi Bakhshi S., Safarzadeh Kozani P. (2022). Nanobody-based CAR-T cells for cancer immunotherapy. Biomark. Res..

[17] Ma H., O’Kennedy R. (2015). The Structure of Natural and Recombinant Antibodies. Methods Mol. Biol..

[18] Chiu M.L., Goulet D.R., Teplyakov A., Gilliland G.L. (2019). Antibody Structure and Function: The Basis for Engineering Therapeutics. Antibodies.

[19] Yanaka S., Yogo R., Kato K. (2020). Biophysical characterization of dynamic structures of immunoglobulin G. Biophys. Rev..

[20] Van de Bovenkamp F.S., Hafkenscheid L., Rispens T., Rombouts Y. (2016). The Emerging Importance of IgG Fab Glycosylation in Immunity. J. Immunol..

[21] Muyldermans S. (2013). Nanobodies: Natural single-domain antibodies. Annu. Rev. Biochem..

[22] Nguyen V.K., Hamers R., Wyns L., Muyldermans S. (2000). Camel heavy-chain antibodies: Diverse germline V(H)H and specific mechanisms enlarge the antigen-binding repertoire. EMBO J..

[23] Nguyen V.K., Desmyter A., Muyldermans S. (2001). Functional heavy-chain antibodies in Camelidae. Adv. Immunol..

[24] Muyldermans S., Cambillau C., Wyns L. (2001). Recognition of antigens by single-domain antibody fragments: The superfluous luxury of paired domains. Trends Biochem. Sci..

[25] Kuroda D., Tsumoto K. (2023). Structural Classification of CDR-H3 in Single-Domain V(H)H Antibodies. Methods Mol. Biol..

[26] Vincke C., Loris R., Saerens D., Martinez-Rodriguez S., Muyldermans S., Conrath K. (2009). General strategy to humanize a camelid single-domain antibody and identification of a universal humanized nanobody scaffold. J. Biol. Chem..

[27] Henry K.A., MacKenzie C.R. (2018). Antigen recognition by single-domain antibodies: Structural latitudes and constraints. MAbs.

[28] Govaert J., Pellis M., Deschacht N., Vincke C., Conrath K., Muyldermans S., Saerens D. (2012). Dual beneficial effect of interloop disulfide bond for single domain antibody fragments. J. Biol. Chem..

[29] Ahmed A.M., Brooks C.L. (2022). X-ray Crystal Structure Analysis of VHH-Protein Antigen Complexes. Methods Mol. Biol..

[30] Huh I., Gene R., Kumaran J., MacKenzie C.R., Brooks C.L. (2014). In situ proteolysis, crystallization and preliminary X-ray diffraction analysis of a VHH that binds listeria internalin B. Acta Crystallogr. F Struct. Biol. Commun..

[31] Sircar A., Sanni K.A., Shi J., Gray J.J. (2011). Analysis and modeling of the variable region of camelid single-domain antibodies. J. Immunol..

[32] Al Qaraghuli M.M., Ferro V.A. (2017). Analysis of the binding loops configuration and surface adaptation of different crystallized single-domain antibodies in response to various antigens. J. Mol. Recognit..

[33] Ikeuchi E., Kuroda D., Nakakido M., Murakami A., Tsumoto K. (2021). Delicate balance among thermal stability, binding affinity, and conformational space explored by single-domain V(H)H antibodies. Sci. Rep..

[34] Cheloha R.W., Harmand T.J., Wijne C., Schwartz T.U., Ploegh H.L. (2020). Exploring cellular biochemistry with nanobodies. J. Biol. Chem..

[35] Dumoulin M., Conrath K., Van Meirhaeghe A., Meersman F., Heremans K., Frenken L.G., Muyldermans S., Wyns L., Matagne A. (2002). Single-domain antibody fragments with high conformational stability. Protein Sci..

[36] Ewert S., Cambillau C., Conrath K., Plückthun A. (2002). Biophysical properties of camelid V(HH) domains compared to those of human V(H)3 domains. Biochemistry.

[37] Liu J.L., Anderson G.P., Delehanty J.B., Baumann R., Hayhurst A., Goldman E.R. (2007). Selection of cholera toxin specific IgNAR single-domain antibodies from a naïve shark library. Mol. Immunol..

[38] Pérez J.M., Renisio J.G., Prompers J.J., van Platerink C.J., Cambillau C., Darbon H., Frenken L.G. (2001). Thermal unfolding of a llama antibody fragment: A two-state reversible process. Biochemistry.

[39] Dolk E., van Vliet C., Perez J.M., Vriend G., Darbon H., Ferrat G., Cambillau C., Frenken L.G., Verrips T. (2005). Induced refolding of a temperature denatured llama heavy-chain antibody fragment by its antigen. Proteins.

[40] Ladenson R.C., Crimmins D.L., Landt Y., Ladenson J.H. (2006). Isolation and characterization of a thermally stable recombinant anti-caffeine heavy-chain antibody fragment. Anal. Chem..

[41] Zabetakis D., Shriver-Lake L.C., Olson M.A., Goldman E.R., Anderson G.P. (2019). Experimental evaluation of single-domain antibodies predicted by molecular dynamics simulations to have elevated thermal stability. Protein Sci..

[42] Goldman E.R., Liu J.L., Zabetakis D., Anderson G.P. (2017). Enhancing Stability of Camelid and Shark Single Domain Antibodies: An Overview. Front. Immunol..

[43] Konning D., Zielonka S., Grzeschik J., Empting M., Valldorf B., Krah S., Schroter C., Sellmann C., Hock B., Kolmar H. (2017). Camelid and shark single domain antibodies: Structural features and therapeutic potential. Curr. Opin. Struct. Biol..

[44] Truong T.T.T., Huynh V.Q., Vo N.T., Nguyen H.D. (2022). Studying the characteristics of nanobody CDR regions based on sequence analysis in combination with 3D structures. J. Genet. Eng. Biotechnol..

[45] Shaw B.F., Schneider G.F., Bilgiçer B., Kaufman G.K., Neveu J.M., Lane W.S., Whitelegge J.P., Whitesides G.M. (2008). Lysine acetylation can generate highly charged enzymes with increased resistance toward irreversible inactivation. Protein Sci..

[46] Bekker G.J., Ma B., Kamiya N. (2019). Thermal stability of single-domain antibodies estimated by molecular dynamics simulations. Protein Sci..

[47] Qiao X., Qu L., Guo Y., Hoshino T. (2021). Secondary Structure and Conformational Stability of the Antigen Residues Making Contact with Antibodies. J. Phys. Chem. B.

[48] Kapp S.J., Larsson I., Van De Weert M., Cárdenas M., Jorgensen L. (2015). Competitive adsorption of monoclonal antibodies and nonionic surfactants at solid hydrophobic surfaces. J. Pharm. Sci..

[49] Dudgeon K., Famm K., Christ D. (2009). Sequence determinants of protein aggregation in human VH domains. Protein Eng. Des. Sel..

[50] Arbabi-Ghahroudi M., To R., Gaudette N., Hirama T., Ding W., MacKenzie R., Tanha J. (2009). Aggregation-resistant VHs selected by in vitro evolution tend to have disulfide-bonded loops and acidic isoelectric points. Protein Eng. Des. Sel..

[51] Liu G., Zhong Q. (2012). Glycation of whey protein to provide steric hindrance against thermal aggregation. J. Agric. Food Chem..

[52] Griffin L.M., Snowden J.R., Lawson A.D., Wernery U., Kinne J., Baker T.S. (2014). Analysis of heavy and light chain sequences of conventional camelid antibodies from Camelus dromedarius and Camelus bactrianus species. J. Immunol. Methods.

[53] Lawrence M.S., Phillips K.J., Liu D.R. (2007). Supercharging proteins can impart unusual resilience. J. Am. Chem. Soc..

[54] Desmyter A., Transue T.R., Ghahroudi M.A., Thi M.H., Poortmans F., Hamers R., Muyldermans S., Wyns L. (1996). Crystal structure of a camel single-domain VH antibody fragment in complex with lysozyme. Nat. Struct. Biol..

[55] Spinelli S., Frenken L.G., Hermans P., Verrips T., Brown K., Tegoni M., Cambillau C. (2000). Camelid heavy-chain variable domains provide efficient combining sites to haptens. Biochemistry.

[56] Saerens D., Pellis M., Loris R., Pardon E., Dumoulin M., Matagne A., Wyns L., Muyldermans S., Conrath K. (2005). Identification of a universal VHH framework to graft non-canonical antigen-binding loops of camel single-domain antibodies. J. Mol. Biol..

[57] Moon D., Tae N., Park Y., Lee S.W., Kim D.H. (2022). Development of Bispecific Antibody for Cancer Immunotherapy: Focus on T Cell Engaging Antibody. Immune Netw..

[58] Suurs F.V., Lub-de Hooge M.N., de Vries E.G.E., de Groot D.J.A. (2019). A review of bispecific antibodies and antibody constructs in oncology and clinical challenges. Pharmacol. Ther..

[59] Schoonjans R., Willems A., Schoonooghe S., Fiers W., Grooten J., Mertens N. (2000). Fab chains as an efficient heterodimerization scaffold for the production of recombinant bispecific and trispecific antibody derivatives. J. Immunol..

[60] Aschmoneit N., Kühl L., Seifert O., Kontermann R.E. (2021). Fc-comprising scDb-based trivalent, bispecific T-cell engagers for selective killing of HER3-expressing cancer cells independent of cytokine release. J. Immunother. Cancer.

[61] Fierle J.K., Brioschi M., de Tiani M., Wetterwald L., Atsaves V., Abram-Saliba J., Petrova T.V., Coukos G., Dunn S.M. (2021). Soluble trivalent engagers redirect cytolytic T cell activity toward tumor endothelial marker 1. Cell Rep. Med..

[62] Joshi K.K., Phung W., Han G., Yin Y., Kim I., Sandoval W., Carter P.J. (2019). Elucidating heavy/light chain pairing preferences to facilitate the assembly of bispecific IgG in single cells. MAbs.

[63] Pekar L., Busch M., Valldorf B., Hinz S.C., Toleikis L., Krah S., Zielonka S. (2020). Biophysical and biochemical characterization of a VHH-based IgG-like bi- and trispecific antibody platform. MAbs.

[64] Sellmann C., Pekar L., Bauer C., Ciesielski E., Krah S., Becker S., Toleikis L., Kügler J., Frenzel A., Valldorf B. (2020). A One-Step Process for the Construction of Phage Display scFv and VHH Libraries. Mol. Biotechnol..

[65] Uchański T., Zögg T., Yin J., Yuan D., Wohlkönig A., Fischer B., Rosenbaum D.M., Kobilka B.K., Pardon E., Steyaert J. (2019). An improved yeast surface display platform for the screening of nanobody immune libraries. Sci. Rep..

[66] Muyldermans S., Baral T.N., Retamozzo V.C., De Baetselier P., De Genst E., Kinne J., Leonhardt H., Magez S., Nguyen V.K., Revets H. (2009). Camelid immunoglobulins and nanobody technology. Vet. Immunol. Immunopathol..

[67] Romao E., Morales-Yanez F., Hu Y., Crauwels M., De Pauw P., Hassanzadeh G.G., Devoogdt N., Ackaert C., Vincke C., Muyldermans S. (2016). Identification of Useful Nanobodies by Phage Display of Immune Single Domain Libraries Derived from Camelid Heavy Chain Antibodies. Curr. Pharm. Des..

[68] Olichon A., de Marco A. (2012). Preparation of a naïve library of camelid single domain antibodies. Methods Mol. Biol..

[69] Liu B., Yang D. (2022). Easily Established and Multifunctional Synthetic Nanobody Libraries as Research Tools. Int. J. Mol. Sci..

[70] Zarebski L.M., Urrutia M., Goldbaum F.A. (2005). Llama single domain antibodies as a tool for molecular mimicry. J. Mol. Biol..

[71] Monegal A., Ami D., Martinelli C., Huang H., Aliprandi M., Capasso P., Francavilla C., Ossolengo G., de Marco A. (2009). Immunological applications of single-domain llama recombinant antibodies isolated from a naïve library. Protein Eng. Des. Sel..

[72] Maass D.R., Sepulveda J., Pernthaner A., Shoemaker C.B. (2007). Alpaca (*Lama pacos*) as a convenient source of recombinant camelid heavy chain antibodies (VHHs). J. Immunol. Methods.

[73] Rothbauer U., Zolghadr K., Tillib S., Nowak D., Schermelleh L., Gahl A., Backmann N., Conrath K., Muyldermans S., Cardoso M.C. (2006). Targeting and tracing antigens in live cells with fluorescent nanobodies. Nat. Methods.

[74] Arbabi Ghahroudi M., Desmyter A., Wyns L., Hamers R., Muyldermans S. (1997). Selection and identification of single domain antibody fragments from camel heavy-chain antibodies. FEBS Lett..

[75] Muyldermans S. (2021). A guide to: Generation and design of nanobodies. FEBS J..

[76] Vincke C., Gutiérrez C., Wernery U., Devoogdt N., Hassanzadeh-Ghassabeh G., Muyldermans S. (2012). Generation of single domain antibody fragments derived from camelids and generation of manifold constructs. Methods Mol. Biol..

[77] Koch-Nolte F., Reyelt J., Schössow B., Schwarz N., Scheuplein F., Rothenburg S., Haag F., Alzogaray V., Cauerhff A., Goldbaum F.A. (2007). Single domain antibodies from llama effectively and specifically block T cell ecto-ADP-ribosyltransferase ART2.2 in vivo. FASEB J..

[78] Romão E., Poignavent V., Vincke C., Ritzenthaler C., Muyldermans S., Monsion B. (2018). Construction of High-Quality Camel Immune Antibody Libraries. Methods Mol. Biol..

[79] Montesi S.B., Désogère P., Fuchs B.C., Caravan P. (2019). Molecular imaging of fibrosis: Recent advances and future directions. J. Clin. Investig..

[80] Zhou Z., Lu Z.R. (2017). Molecular imaging of the tumor microenvironment. Adv. Drug Deliv. Rev..

[81] Guo R., Meng X., Wang F., Yu J., Xie Q., Zhao W., Zhu L., Kung H.F., Yang Z., Li N. (2021). ^68^Ga-P15-041, a Novel Bone Imaging Agent for Diagnosis of Bone Metastases. Front. Oncol..

[82] Oh J.R., Byun B.H., Hong S.P., Chong A., Kim J., Yoo S.W., Kang S.R., Kim D.Y., Song H.C., Bom H.S. (2011). Comparison of ^131^I whole-body imaging, ^131^I SPECT/CT, and ^18^F-FDG PET/CT in the detection of metastatic thyroid cancer. Eur. J. Nucl. Med. Mol. Imaging.

[83] Iravani A., Hicks R.J. (2020). Imaging the Cancer Immune Environment and Its Response to Pharmacologic Intervention, Part 1: The Role of ^18^F-FDG PET/CT. J. Nucl. Med..

[84] Huang L., Gainkam L.O., Caveliers V., Vanhove C., Keyaerts M., De Baetselier P., Bossuyt A., Revets H., Lahoutte T. (2008). SPECT imaging with ^99m^Tc-labeled EGFR-specific nanobody for in vivo monitoring of EGFR expression. Mol. Imaging Biol..

[85] Berland L., Kim L., Abousaway O., Mines A., Mishra S., Clark L., Hofman P., Rashidian M. (2021). Nanobodies for Medical Imaging: About Ready for Prime Time?. Biomolecules.

[86] Gainkam L.O., Keyaerts M., Caveliers V., Devoogdt N., Vanhove C., Van Grunsven L., Muyldermans S., Lahoutte T. (2011). Correlation between epidermal growth factor receptor-specific nanobody uptake and tumor burden: A tool for noninvasive monitoring of tumor response to therapy. Mol. Imaging Biol..

[87] Warnders F.J., Terwisscha van Scheltinga A.G.T., Knuehl C., van Roy M., de Vries E.F.J., Kosterink J.G.W., de Vries E.G.E., Lub-de Hooge M.N. (2017). Human Epidermal Growth Factor Receptor 3-Specific Tumor Uptake and Biodistribution of ^89^Zr-MSB0010853 Visualized by Real-Time and Noninvasive PET Imaging. J. Nucl. Med..

[88] Ingram J.R., Dougan M., Rashidian M., Knoll M., Keliher E.J., Garrett S., Garforth S., Blomberg O.S., Espinosa C., Bhan A. (2017). PD-L1 is an activation-independent marker of brown adipocytes. Nat. Commun..

[89] Liu Q., Jiang L., Li K., Li H., Lv G., Lin J., Qiu L. (2021). Immuno-PET imaging of ^68^Ga-labeled nanobody Nb109 for dynamic monitoring the PD-L1 expression in cancers. Cancer Immunol. Immunother..

[90] Li D., Cheng S., Zou S., Zhu D., Zhu T., Wang P., Zhu X. (2018). Immuno-PET Imaging of ^89^Zr Labeled Anti-PD-L1 Domain Antibody. Mol. Pharm..

[91] Broos K., Keyaerts M., Lecocq Q., Renmans D., Nguyen T., Escors D., Liston A., Raes G., Breckpot K., Devoogdt N. (2017). Non-invasive assessment of murine PD-L1 levels in syngeneic tumor models by nuclear imaging with nanobody tracers. Oncotarget.

[92] Krasniqi A., D’Huyvetter M., Xavier C., Van der Jeught K., Muyldermans S., Van Der Heyden J., Lahoutte T., Tavernier J., Devoogdt N. (2017). Theranostic Radiolabeled Anti-CD20 sdAb for Targeted Radionuclide Therapy of Non-Hodgkin Lymphoma. Mol. Cancer Ther..

[93] Wang C., Chen Y., Hou Y.N., Liu Q., Zhang D., Zhao H., Zhang Y., An S., Li L., Hou J. (2021). ImmunoPET imaging of multiple myeloma with [^68^Ga]Ga-NOTA-Nb1053. Eur. J. Nucl. Med. Mol. Imaging.

[94] Rashidian M., Ingram J.R., Dougan M., Dongre A., Whang K.A., LeGall C., Cragnolini J.J., Bierie B., Gostissa M., Gorman J. (2017). Predicting the response to CTLA-4 blockade by longitudinal noninvasive monitoring of CD8 T cells. J. Exp. Med..

[95] Zhao H., Wang C., Yang Y., Sun Y., Wei W., Wang C., Wan L., Zhu C., Li L., Huang G. (2021). ImmunoPET imaging of human CD8^+^ T cells with novel ^68^Ga-labeled nanobody companion diagnostic agents. J. Nanobiotechnol..

[96] Vaneycken I., Govaert J., Vincke C., Caveliers V., Lahoutte T., De Baetselier P., Raes G., Bossuyt A., Muyldermans S., Devoogdt N. (2010). In vitro analysis and in vivo tumor targeting of a humanized, grafted nanobody in mice using pinhole SPECT/micro-CT. J. Nucl. Med..

[97] Chatalic K.L., Veldhoven-Zweistra J., Bolkestein M., Hoeben S., Koning G.A., Boerman O.C., de Jong M., van Weerden W.M. (2015). A Novel ^111^In-Labeled Anti-Prostate-Specific Membrane Antigen Nanobody for Targeted SPECT/CT Imaging of Prostate Cancer. J. Nucl. Med..

[98] Vosjan M.J., Vercammen J., Kolkman J.A., Stigter-van Walsum M., Revets H., van Dongen G.A. (2012). Nanobodies targeting the hepatocyte growth factor: Potential new drugs for molecular cancer therapy. Mol. Cancer Ther..

[99] Klarenbeek A., El Mazouari K., Desmyter A., Blanchetot C., Hultberg A., de Jonge N., Roovers R.C., Cambillau C., Spinelli S., Del-Favero J. (2015). Camelid Ig V genes reveal significant human homology not seen in therapeutic target genes, providing for a powerful therapeutic antibody platform. MAbs.

[100] Sang Z., Xiang Y., Bahar I., Shi Y. (2021). Llamanade: An open-source computational pipeline for robust nanobody humanization. bioRxiv.

[101] Xavier C., Vaneycken I., D’Huyvetter M., Heemskerk J., Keyaerts M., Vincke C., Devoogdt N., Muyldermans S., Lahoutte T., Caveliers V. (2013). Synthesis, preclinical validation, dosimetry, and toxicity of 68Ga-NOTA-anti-HER2 Nanobodies for iPET imaging of HER2 receptor expression in cancer. J. Nucl. Med..

[102] Keyaerts M., Xavier C., Heemskerk J., Devoogdt N., Everaert H., Ackaert C., Vanhoeij M., Duhoux F.P., Gevaert T., Simon P. (2016). Phase I Study of 68Ga-HER2-Nanobody for PET/CT Assessment of HER2 Expression in Breast Carcinoma. J. Nucl. Med..

[103] Kijanka M., Dorresteijn B., Oliveira S., van Bergen en Henegouwen P.M. (2015). Nanobody-based cancer therapy of solid tumors. Nanomedicine.

[104] Roovers R.C., Laeremans T., Huang L., De Taeye S., Verkleij A.J., Revets H., de Haard H.J., van Bergen en Henegouwen P.M. (2007). Efficient inhibition of EGFR signaling and of tumour growth by antagonistic anti-EFGR Nanobodies. Cancer Immunol. Immunother..

[105] Roskoski R. (2014). The ErbB/HER family of protein-tyrosine kinases and cancer. Pharmacol. Res..

[106] Omidfar K., Rasaee M.J., Modjtahedi H., Forouzandeh M., Taghikhani M., Golmakani N. (2004). Production of a novel camel single-domain antibody specific for the type III mutant EGFR. Tumour Biol..

[107] Roovers R.C., Vosjan M.J., Laeremans T., el Khoulati R., de Bruin R.C., Ferguson K.M., Verkleij A.J., van Dongen G.A., van Bergen en Henegouwen P.M. (2011). A biparatopic anti-EGFR nanobody efficiently inhibits solid tumour growth. Int. J. Cancer.

[108] Tijink B.M., Laeremans T., Budde M., Stigter-van Walsum M., Dreier T., de Haard H.J., Leemans C.R., van Dongen G.A. (2008). Improved tumor targeting of anti-epidermal growth factor receptor Nanobodies through albumin binding: Taking advantage of modular Nanobody technology. Mol. Cancer Ther..

[109] Harwood S.L., Alvarez-Cienfuegos A., Nuñez-Prado N., Compte M., Hernández-Pérez S., Merino N., Bonet J., Navarro R., Van Bergen En Henegouwen P.M.P., Lykkemark S. (2017). ATTACK, a novel bispecific T cell-recruiting antibody with trivalent EGFR binding and monovalent CD3 binding for cancer immunotherapy. Oncoimmunology.

[110] Luker K.E., Gupta M., Steele J.M., Foerster B.R., Luker G.D. (2009). Imaging ligand-dependent activation of CXCR7. Neoplasia.

[111] Rajagopal S., Kim J., Ahn S., Craig S., Lam C.M., Gerard N.P., Gerard C., Lefkowitz R.J. (2010). Beta-arrestin- but not G protein-mediated signaling by the “decoy” receptor CXCR7. Proc. Natl. Acad. Sci. USA.

[112] Wang J., Shiozawa Y., Wang J., Wang Y., Jung Y., Pienta K.J., Mehra R., Loberg R., Taichman R.S. (2008). The role of CXCR7/RDC1 as a chemokine receptor for CXCL12/SDF-1 in prostate cancer. J. Biol. Chem..

[113] Miao Z., Luker K.E., Summers B.C., Berahovich R., Bhojani M.S., Rehemtulla A., Kleer C.G., Essner J.J., Nasevicius A., Luker G.D. (2007). CXCR7 (RDC1) promotes breast and lung tumor growth in vivo and is expressed on tumor-associated vasculature. Proc. Natl. Acad. Sci. USA.

[114] Shakir M., Tang D., Zeh H.J., Tang S.W., Anderson C.J., Bahary N., Lotze M.T. (2015). The chemokine receptors CXCR4/CXCR7 and their primary heterodimeric ligands CXCL12 and CXCL12/high mobility group box 1 in pancreatic cancer growth and development: Finding flow. Pancreas.

[115] Low S., Wu H., Jerath K., Tibolla A., Fogal B., Conrad R., MacDougall M., Kerr S., Berger V., Dave R. (2020). VHH antibody targeting the chemokine receptor CX3CR1 inhibits progression of atherosclerosis. MAbs.

[116] Jähnichen S., Blanchetot C., Maussang D., Gonzalez-Pajuelo M., Chow K.Y., Bosch L., De Vrieze S., Serruys B., Ulrichts H., Vandevelde W. (2010). CXCR4 nanobodies (VHH-based single variable domains) potently inhibit chemotaxis and HIV-1 replication and mobilize stem cells. Proc. Natl. Acad. Sci. USA.

[117] Maussang D., Mujić-Delić A., Descamps F.J., Stortelers C., Vanlandschoot P., Stigter-van Walsum M., Vischer H.F., van Roy M., Vosjan M., Gonzalez-Pajuelo M. (2013). Llama-derived single variable domains (nanobodies) directed against chemokine receptor CXCR7 reduce head and neck cancer cell growth in vivo. J. Biol. Chem..

[118] Weis W.I., Kobilka B.K. (2018). The Molecular Basis of G Protein-Coupled Receptor Activation. Annu. Rev. Biochem..

[119] Bobkov V., Arimont M., Zarca A., De Groof T.W.M., van der Woning B., de Haard H., Smit M.J. (2019). Antibodies Targeting Chemokine Receptors CXCR4 and ACKR3. Mol. Pharmacol..

[120] McMahon C., Staus D.P., Wingler L.M., Wang J., Skiba M.A., Elgeti M., Hubbell W.L., Rockman H.A., Kruse A.C., Lefkowitz R.J. (2020). Synthetic nanobodies as angiotensin receptor blockers. Proc. Natl. Acad. Sci. USA.

[121] Obeng E.M., Dzuvor C.K.O., Danquah M.K. (2022). Anti-SARS-CoV-1 and -2 nanobody engineering towards avidity-inspired therapeutics. Nano Today.

[122] Eichhoff A.M., Börner K., Albrecht B., Schäfer W., Baum N., Haag F., Körbelin J., Trepel M., Braren I., Grimm D. (2019). Nanobody-Enhanced Targeting of AAV Gene Therapy Vectors. Mol. Ther. Methods Clin. Dev..

[123] Moradi A., Pourseif M.M., Jafari B., Parvizpour S., Omidi Y. (2020). Nanobody-based therapeutics against colorectal cancer: Precision therapies based on the personal mutanome profile and tumor neoantigens. Pharmacol. Res..

[124] Wade J., Rimbault C., Ali H., Ledsgaard L., Rivera-de-Torre E., Abou Hachem M., Boddum K., Mirza N., Bohn M.F., Sakya S.A. (2022). Generation of Multivalent Nanobody-Based Proteins with Improved Neutralization of Long α-Neurotoxins from Elapid Snakes. Bioconjug. Chem..

[125] Cortez-Retamozo V., Lauwereys M., Hassanzadeh Gh G., Gobert M., Conrath K., Muyldermans S., De Baetselier P., Revets H. (2002). Efficient tumor targeting by single-domain antibody fragments of camels. Int. J. Cancer.

[126] Hu Y., Liu C., Muyldermans S. (2017). Nanobody-Based Delivery Systems for Diagnosis and Targeted Tumor Therapy. Front. Immunol..

[127] Jovčevska I., Muyldermans S. (2020). The Therapeutic Potential of Nanobodies. BioDrugs.

[128] Overington J.P., Al-Lazikani B., Hopkins A.L. (2006). How many drug targets are there?. Nat. Rev. Drug Discov..

